# Micronucleus Test of Kong-Jin-Dan, a Polyherbal Formula, in Bone Marrow Cells of Male ICR Mice

**DOI:** 10.5487/TR.2008.24.3.213

**Published:** 2008-09-01

**Authors:** Sang-Nam Lee, Ji-Ha Park, Sae-Kwang Ku

**Affiliations:** 19 Department of Anatomy and Histology, College of Oriental Medicine, Daegu Haany University, 290, Yugok-dong, Gyeongsan-si, Gyeongsangbuk-do, 712-715 Korea; 29 Development Team for The New Drug of Oriental Medicine (BK21 program), Daegu Haany University, Gyeongsan, 712-715 Korea

**Keywords:** Kong-Jin-Dan, β-Glucan, Micronucleus test, Genotoxicity, Mice, White blood cells

## Abstract

In this research, the genotoxic effects of Kong-Jin-Dan (KJD), a polyherbal formula were evaluated using the mouse micronucleus test. KJD was administered once a day for 2 continuous days by oral gavage to male ICR mice at doses of 2000, 1000 and 500 mg/kg. Cyclophosphamide was used as a known genotoxic agent in a positive control. The appearance of a micronucleus is used as an index for genotoxic potential. In addition, the changes on the total white blood cells and differential counts on the lymphocytes, neutrophils, eosinophils, basophils and monocytes in the prepared blood smears were also conducted to observe the possible immunosuppress. The results obtained indicated that KJD shows no genotoxicity effects up to 2000 mg/kg dosing levels, but KJD shows slight increased trends in the blood total leukocyte numbers as pharmacological effects of immune stimulation. In addition, it is also considered that there were no problems from cytotoxicity of KJD tested in this study because the polychromatic erythrocyte ratio was detected as > 0.42 in all tested groups.

## INTRODUCTION

A polyherbal formula, Kong-Jin-Dan (KJD) is one of the most famous tonic agents, in Korean traditional medicine, and consisted of 4 herbs including Angelicae gigas radix, Ginseng steamed red, Corni fructus and Rehmanniae radix preparata, and 2 animal resources - antler and musk. These 6 agents were plastered using honey, and coated by gold plates. The hypolipemic and immune stimulatory effects of KJD are relatively well documented (Kim and Bae, 1989; Kim *et al.*, 1999).

As increase of the concern in the functional food and well being in life, the demands and consumption of functional food originated form natural sources are increased (Lee *et al.*, 2003). However, the toxicological aspects about these natural origin-functional foods has been neglected because of the reasons that they has been used as various purpose for long times. Therefore, it is considered that more detailed and systemic toxicological studies should be performed to control the abuse and potential toxicities even if they have been used as traditional folk medicine. The toxicological studies about KJD also have been neglected except for only basic single dose toxicities in mice (Park *et al.*, 2007). There are no available genotoxic studies about KJD.

Bone marrow cytogenetics, micronucleus test is a useful short-term technique for elucidating the mechanism as well as to identify the substances for their clastogenic and anticlastogenic activity (Renner, 1990). In Korea Food and Drug Administration (KFDA) guideline (2005-60, 2005), the genotoxicity should be tested prior to develop a new drug even though they have natural origin. Most of the mixtures having natural origin, genotoxicity has been performed using *in vivo* like micronucleus test (Kalantari *et al.*, 2007).

The objective of the present study, therefore, was to obtain the genotoxic information about KJD, a polyherbal formula having various pharmacological effects with the effects on the total white blood cells and their differential counts in the prepared blood to observe the possible immunosuppression, and further clarify their safety for clinical use.

## MATERIALS AND METHODS

**Animals and husbandry.** Thirty-five male ICR mice (6-week old upon receipt, SLC, Japan) were used after acclimatization for 6 days. The body weights of animals at receipt are ranged in 29~30 g. Animals were allocated seven per polycarbonate cage in a temperature (20~25°C) and humidity (30~35%) controlled room. Light : dark cycle was 12 h : 12 h and feed (Samyang, Korea) and water were supplied free to access. Animals were marked by picric acid. The experimental protocols were conducted in accordance with internationally accepted principles for laboratory animal use and care as found in the Korea Food and Drug Administration (KFDA) guidelines.

**Test articles and formulation.** KJD used in this study was purchased from Daegu Oriental Hospital of Daegu Haany University (Daegu, Korea) as listed in Table 1. All 8 types of individual components purchase from Human herbs (Daegu, Korea). Briefly, approximate amounts, listed in Table 1 of antler, Angelicae gigantis radix, Ginseng steamed red, Corni fructus, Rehmanniae radix preparata and musk were grinded, and then mixed with honey. After that, they were coated with gold plate. Deep brown gold-coated plasters, KJD was stored in a refrigerator at -20°C to protect from light and degeneration. It was well suspended up to 200 mg/ml concentration levels and appeared to be a deep brown homogenous suspension. The test article was orally administered at a dosage volume of 10 ml/kg, once a day for 2 days by oral gavage to mice; total 2000, 1000 and 500 mg/kg using distilled water as vehicle. Cyclophosphamide·$H_2O$ (CPA; Sigma, USA) was used as an identified genotoxic agents in a positive control group. CPA was dissolved in saline and once intraperitoneally administered at a volume of 10 ml/kg.

**Grouping and dosing.** The animals were allocated into five groups 7 mice each. The fixed highest dosage level of 2000 mg/kg oral dosing was chosen in accordance to the results of single dose toxicity test in mice (Park *et al.*, 2007), and 500 and 250 mg/kg was selected using the common ratio 2 according to the KFDA guidelines (2005). Control negative (taken vehicle) and control positive (CPA; 70 mg/kg-single treatment) were included by recommendation of KFDA guidelines (2005) and Organization for Economic Co-Operation and Development (OECD) guidelines (1997). The administered doses and schedule of these drugs are listed in Table 2.

**Observation of clinical signs.** All abnormal clinical signs were recorded before and after dosing at least twice a day based on the functional observational battery test (Irwin, 1968; Dourish, 1987).

**Table 1.** Composition of Kong-Jin-Dan used in this study

| Herbs | Scientific Names | Source | Amounts (g/pill) |
|---|---|---|---|
| Antler (Cornus cervi parvum) | *Cervus elaphus* Linne | Russia | 0.683 |
| Angelicae gigantis radix | *Angelica gigas* Nakai | Korea | 0.683 |
| Ginseng steamed red | *Panax ginseng* CA Mey. | Korea | 0.683 |
| Corni fructus | *Cornus officinalis* Sieb. Et Zucc | Korea | 0.683 |
| Rehmanniae radix preparata | *Rehmannia glutinosa* (Gaertner) Liboschitz | Korea | 0.683 |
| Musk | *Moschus moschiferus* Linne | Russia | 0.122 |
| Honey | *Apis indica* Radoszkowski | Korea | 2.506 |
| Gold plate | | Korea | 0.006 |
| Total | 8 types | | 6.050 |

Kong-Jin-Dan used in this study was purchased from Daegu Oriental Hospital of Daegu Hanny University (Daegu, Korea), and all 8 types of individual components were purchased from Human Herbs (Daegu, Korea).

**Table 2.** Experimental design used in this study

| Group | Dosing materials (route) | No. of animals | Animal No. | Total dose (mg/kg) |
|---|---|---|---|---|
| G0* | Distilled water (oral) | 7 | G0-01~G0-07 | 0 |
| G1** | CPA (intraperitoneal) | 7 | G1-01~G1-07 | 70 |
| G2 | Kong-Jin-Dan (oral) | 7 | G2-01~G2-07 | 2000 |
| G3 | Kong-Jin-Dan (oral) | 7 | G3-01~G3-07 | 1000 |
| G4 | Kong-Jin-Dan (oral) | 7 | G4-01~G4-07 | 500 |

*Vehicle control; **Positive control; All test articles were taken once a day for coupled days; CPA, cyclophosphamide.

**Body weight changes.** Body weights were measured once a day.

**Bone marrow preparation.** All animals were sacrificed 24 h post administration using carbon dioxide, and bilateral femur was separated. Bone marrow preparations were made according to Schimid (1975). In brief, bone marrow cells were collected from aforementioned femur in 3 ml of inactivated fetal bovine serum (Gibco BRL, USA), centrifuged, and smeared on slides. Preparations were dried, and fixed by submerging in absolute methanol (for 10~20 min). Fixed slides were stained as follows;

| | |
|---|---|
| May-Grunwald stain | 3 min |
| May-Grunwald stain (1 : 1 diluted) | 2 min |
| Giemsa stain (1 : 6 diluted) | 10 min |

**Observation and recoding of micronuclei.** Slides were randomly coded and examined under × 1000 magnification by two different experts. Small round or oval shaped bodies, size of which ranging from 1/5 to 1/20 diameter of polychromatic erythrocytes (PCE), were counted as micronuclei (MN). Attention was given to discriminate micronuclei from artifacts (Fig. 1). Results were expressed as the number of MNPCEs in 2000 PCEs. Mean number of MNPCE ± standard deviation was calculated for each treatment group. In addition, PCE/(PCE + normochromatic erythrocytes (NCE)) ratio were also calculated by counting 500 erythrocytes, for detecting the possibility of cytotoxicity (Heddle *et al.*, 1984).

**Blood collection and leukocyte counts.** Blood were collected at sacrifice from vena cava, and total blood leukocyte numbers were calculated using counting chamber, diluting pipette and Türk solution as dilution solution. In addition, cell numbers of lymphocytes, neutrophils, eosinophils, basophils and monocytes were calculated among 100 total leukocytes in smear blood samples stained with Giemsa (Fig. 1).

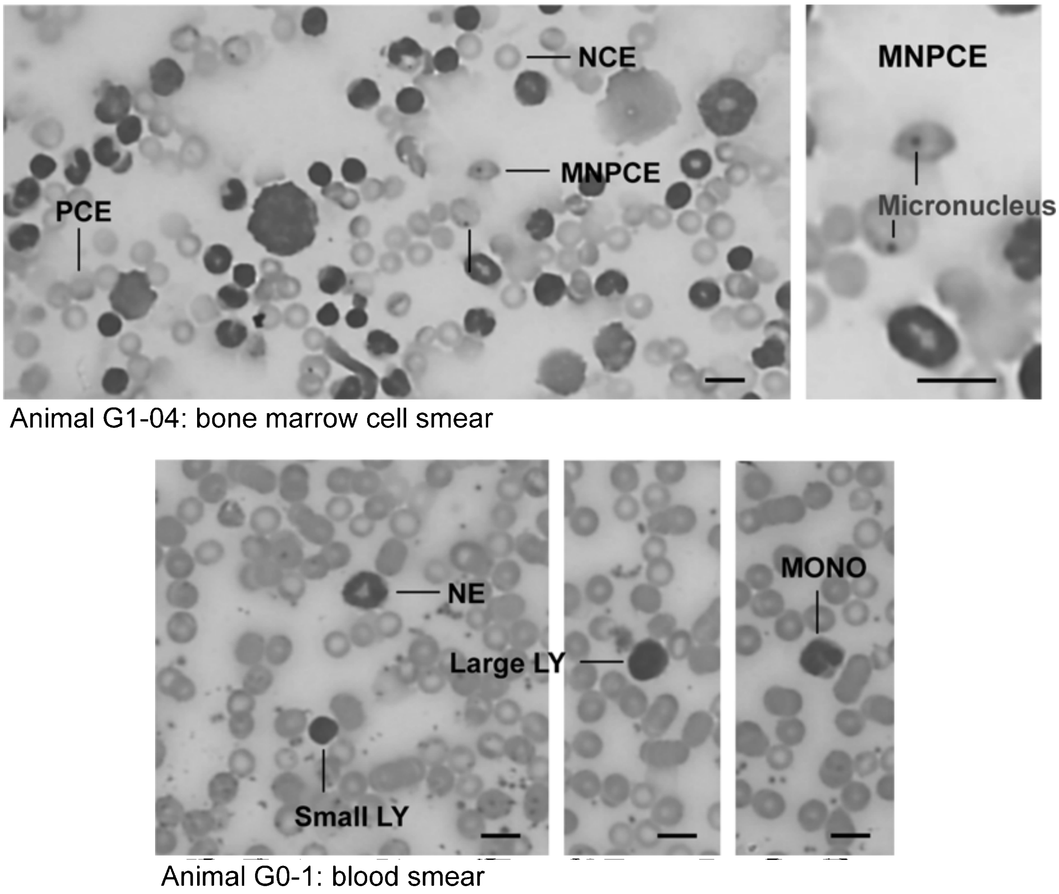

**Fig. 1.** Representative cytology of bone marrow cell and blood cell smears. In prepared bone marrow cell smear, polychromatic erythrocyte (PCE), normochromatic erythrocyte (NCE), PCE with one or more nuclei (MNPCE) were counted based on the morphology. NCEs containing nucleus were not calculated. In prepared blood smear, neutrophils (NE), eosinophils, basophils, moncytes (MONO) and lymphocytes (LY) were counted based on the morphology. Scale bars = 10 μm.

**Statistical analyses.** Multiple comparison tests for different dose groups were conducted. Variance homogeneity was examined using the Levene test. If the Levene test indicated no significant deviations from variance homogeneity, the obtain data were analyzed by one way ANOVA test followed by Tukey HSD test to determine which pairs of group comparison were significantly different. In case of significant deviations from variance homogeneity were observed at Levene test, a non-parametric comparison test, the Mann-Whitney U-Wilcoxon Rank Sum W test was conducted to determine the specific pairs of group comparison, which are significantly different. The result of statistical evaluation was regarded significantly when the P value was less than 0.05. In addition, the study was accepted when all of the PCE/(PCE + NCE) ratio are greater than 0.20 (Heddle *et al.*, 1984). Statistical analyses were carried out using SPSS for Windows (Release 6.1.3., SPSS Inc., USA).

## RESULTS

**Mortalities.** No test article-treatment related unscheduled mortalities were detected in all tested doses during the observation periods.

**Clinical signs.** During the observation period, no abnormal clinical signs were observed from KJD-treatment.

**Body weight changes.** No meaningful changes on body weights were detected in CPA and all the tested doses of KJD treated groups as compared to that of control negative group (taken vehicle only) (Table 3).

**Changes on MNPCE numbers and PCE ratio.** Significantly ($p < 0.01$) increase of number of MNPCEs among 2000 PCEs was detected in CPA 70 mg/kg a positive control group. However, no significant changes on MNPCE numbers were detected in all three different KJD treated groups tested as compared with vehicle control (Table 4). The PCE/(PCE + NCE) ratio in total 500 erythrocytes was detected above 0.42 in all tested groups including negative and positive control (Table 4).

**Changes on the blood leukocytes.** Except for non-significant decreases of blood total leukocyte numbers detected in CPA-injected group and increased trends in all three different dose of KJD groups as compared with intact control, no meaningful changes on the total blood leukocyte numbers and their differential counts were observed in all tested groups as compared with intact control, respectively (Table 5).

## DISCUSSION

Micronucleus assays were first introduced in the early

**Table 3.** Changes in the body weights

| Group[1)] | | Day after dosing | | |
|---|---|---|---|---|
| | | Day 0[2)] | Day 1 | At a termination |
| Male | G0 | 35.07 ± 1.49 | 34.50 ± 2.09 | 31.80 ± 1.37 |
| ICR | G1 | 35.21 ± 1.15 | 35.36 ± 1.10 | 32.43 ± 0.85 |
| Mice | G2 | 34.46 ± 1.24 | 34.19 ± 1.25 | 31.10 ± 0.94 |
| | G3 | 34.97 ± 1.57 | 35.03 ± 1.56 | 31.57 ± 1.49 |
| | G4 | 34.87 ± 0.93 | 35.09 ± 1.09 | 32.01 ± 0.77 |

[a]Values are expressed as mean ± SD, g of 7 mice; [1)]Groups were listed in Table 1; [2)]Start day of test article administration; All animals were overnight fasted at Day 0 and a termination, respectively.

**Table 4.** Changes in MNPCE numbers and PCE/(PCE + NCE) ratio observed in mice

| Group[1)] | | MNPCEs/2000 PCEs | PCE/(PCE + NCE) ratio PCE + NCE = 500 cells |
|---|---|---|---|
| Male | G0 | 0.71 ± 0.76 | 0.47 ± 0.02 |
| ICR | G1 | 79.00 ± 6.40* | 0.42 ± 0.04 |
| Mice | G2 | 0.57 ± 0.79 | 0.47 ± 0.02 |
| | G3 | 0.71 ± 1.11 | 0.48 ± 0.03 |
| | G4 | 0.57 ± 0.79 | 0.47 ± 0.02 |

Values are expressed as mean ± SD of 7 mice; [1)]Groups were listed in Table 1; *$p < 0.01$ compared to that of G0 by Mann-Whitney U-Wilcoxon Rank Sum W test.

**Table 5.** Changes in the blood leukocytes: total leukocyte numbers and differential counts of lymphocytes, neutrophils, eosinophils, basophils and monocytes observed in blood smear prepared

| Group[1)] | | Total leukocyte numbers ($\times 10^3$ cells/$mm^3$) | Proportions among 100 leukocytes (%) | | | | |
|---|---|---|---|---|---|---|---|
| | | | Lymphocytes | Neutrophils | Eosinophils | Basophils | Monocytes |
| Male | G0 | 2.47 ± 0.69 | 81.57 ± 6.13 | 17.43 ± 6.40 | 0.00 ± 0.00 | 0.00 ± 0.00 | 1.00 ± 1.00 |
| ICR | G1 | 1.96 ± 0.64 | 81.14 ± 8.38 | 16.71 ± 7.59 | 0.00 ± 0.00 | 0.00 ± 0.00 | 2.14 ± 1.57 |
| Mouse | G2 | 3.29 ± 1.23 | 82.43 ± 4.61 | 17.00 ± 4.36 | 0.00 ± 0.00 | 0.00 ± 0.00 | 0.57 ± 0.98 |
| | G3 | 2.81 ± 0.62 | 82.43 ± 5.68 | 16.71 ± 6.18 | 0.00 ± 0.00 | 0.00 ± 0.00 | 0.86 ± 0.69 |
| | G4 | 2.81 ± 0.76 | 83.00 ± 6.03 | 15.57 ± 6.55 | 0.00 ± 0.00 | 0.00 ± 0.00 | 1.43 ± 1.40 |

Values are expressed as mean ± SD of 7 mice; [1)]Groups were listed in Table 1.

1970's for the examination of genotoxic activity of chemical agents (Matter and Schmid, 1971; Heddle, 1973). The procedure is based on the observation that mitotic cells with chromatid breaks or incomplete exchanges or with malfunction of the spindle apparatus suffer from disturbances in anaphase distribution of their chromatin. After telophase, a sizable portion of this displaced chromatin is not included in the nuclei of the daughter cells but forms single or multiple micronuclei in the cell cytoplasm. The frequency of the appearance of micronuclei depends both upon the rate of chromosome breakage or loss and the rate of cell division (Von Ledebur and Schmid, 1973; Heddle *et al.*, 1984). Although micronuclei can occur in almost all dividing cells, mouse bone marrow is usually the tissue used for the micronucleus test, and any agent which induces chromosomal aberrations can also produce micronuclei (Heddle *et al.*, 1983, 1984).

Because of its simplicity and efficacy, the micronucleus test has become a popular and useful *in vivo* procedure for the detection of chemically-induced chromosome damage. The number of reports from micronucleus testing has increased dramatically in the scientific literature during the past decade (Ashby, 1985), and the value of this test for examining the mutagenicity and carcinogenicity of chemicals has been emphasized, particularly when it is used in combination with other cytogenetic assays (Heddle *et al.*, 1984).

In the present study, the genotoxic effects of KJD were evaluated using the mouse micronucleus test with effects on the blood total leukocyte numbers and their differential counts. As the results obtained in the present study, KJD shows no genotoxicity effect up to 2000 mg/kg dosing levels. The highest dosage used in the present study was selected as 2000 mg/kg, based on the results of single dose toxicity test in mice (Park *et al.*, 2007), and vehicle and positive control were added according to the recommendation of KFDA (2005) and OECD (1997) guidelines. KJD shows non-significant increase trends of the blood total leukocyte numbers in all three dosed levels, it means KJD has immune stimulatory effects as previous reports (Kim *et al.*, 1999), not a toxicological change.

The PCE/(PCE + NCE) was used as index of cytotoxicity and the study was accepted when all of the PCE/(PCE + NCE) ratio are greater than 0.20 (Heddle *et al.*, 1984). The PCE/(PCE + NCE) ratio was detected as > 0.42 in all tested groups including negative and positive control in the present study. That is no problem from cytotoxicity of the tested articles used in this work.

CPA is a widely used anti-neoplasic drug, employed either alone or in combination with other products (Grochow, 1996). The parent drug is biologically inactive, however after biotransformation by microsomal enzymes a number of active metabolites capable of alkylating nucleic acids (Miyauchi *et al.*, 1990), damage the chromosomes (through generation of free-radicals) and/or alkylating the DNA thereby producing mutagenicity (El-Bayoumy, 2001) were produced. In the present study, CPA used as a positive control, and it showed a significant increases of MNPCE ratios. This indicates that the experiment protocol and the results of the present study are acceptable, and no meaningful increases of MNPCE were reported up to 2000 mg/kg of KJD.

Based on the results, it is concluded that KJD shows no genotoxicity and immunosuppress effects up to 2000 mg/kg dosing levels. In addition, it is also considered that there were no problems from cytotoxicity of Polycan because the polychromatic erythrocyte ratio was estimated as > 0.42 in all tested groups.

## References

[CR1] Ashby J (1985). Is there a continuing role for the intraperitoneal injection route of exposure in short-term rodent genotoxicity assays?. Mutat. Res..

[CR2] Dourish, C.T. (1987). Effects of drugs on spontaneous motor activity in experimental psychopharmacology (Greenshaw A.J. and Dourish, C.T., Eds.), Humana Press, Clifton, pp. 325–334.

[CR3] El-Bayoumy K (2001). The protective role of selenium on genetic damage and on cancer. Mutat. Res..

[CR4] Grochow, L.B. (1996). Covalent-DNA binding drugs in The chemotherapy source book (Perry, M.C., Ed.), Williams & Wilkins, Baltimore, pp. 293–316.

[CR5] Heddle JA (1973). A rapid *in vivo* test for chromosome damage. Mutat. Res..

[CR6] Heddle JA, Hite M, Kirkhart B, Mavournin K, MacGregor JT, Newell GW, Salamone MF (1983). The induction of micronuclei as a measure of genotoxicity: a report of the U.S. environmental protection agency genetox program. Mutat. Res..

[CR7] Heddle John A., Stuart Earl, Salamone Michael F. (1984). THE BONE MARROW MICRONUCLEUS TEST. Handbook of Mutagenicity Test Procedures.

[CR8] Kalantari H, Larki A, Latifi SM (2007). The genotoxicity study of garlic and pasipy herbal drops by peripheral blood micronucleus test. Acta Physiol. Hung.

[CR9] Kim YH, Bae MJ (2001). The effect of Kong Jin Dan on the lipid metabolism in rats fed high fat-diet. J. East-West Med..

[CR10] Kim YS, Lee JD, Kim CH, Choi DY, Park DS, Nam SS, Kang SK (1999). The Effect of Kongjindan Aqua-acupuncture on the CD4+ T cell count at al. in rats were administered with MTX. J. Korean Acup. Moxi. Soc..

[CR11] Korea F, Drug Administration (2005). Testing Guidelines for Safety Evaluation of Drugs.

[CR12] Lee JE, Kim HJ C E, Chai HY, Yun YW, Kim DJ, Nam SY, Lee BJ, Ahn BW, Kang HG, Kim YB (2003). Four-week repeated-dose toxicity study on Pinellia Extract. Korean J. Lab. Anim. Sci..

[CR13] Matter B, Schmid W (1971). Trenimon-induced chromosomal damage in bone-marrow cells of six mammalian species, evaluated by the micronucleus test. Mutat. Res.

[CR14] Miyauchi A, Hiramine C, Tanaka S, Hojo K (1990). Differential effects of a single dose of cyclophosphamide on T cell subsets of the thymus and spleen in mice: flow cytofluorometry analysis. Tohoku J. Exp. Med..

[CR15] Organization for Economic Co-OperationDevelopment. (1997). Guideline for the Testing of Chemicals TG No. 474.

[CR16] Irwin S (1968). Comprehensive observational assessment: Ia. A systemic, quantitative procedure for assessing the behavioral and physiological state of the mouse. Psychopharmacology.

[CR17] Park MY, Choi HY, Kim JD, Lee HS, Ku SK (2007). Single oral dose toxicity test of Kong-Jin-Dan, a polyherbal formula in ICR mice. J. Appl. Pharmacology.

[CR18] Renner HW (1990). *In vivo* effects of single or combined dietary antimutagens on mutagen-induced chromosomal aberrations. Mutat. Res..

[CR19] Schimid W (1975). The Micronucleus Test. Mutat. Res.

[CR20] Von Ledebur M, Schmid W (1973). The micronucleus test - methodological aspects. Mutat. Res..

